# Strategic Analysis of Participants in the Provision of Elderly Care Services—An Evolutionary Game Perspective

**DOI:** 10.3390/ijerph18168595

**Published:** 2021-08-14

**Authors:** Jiahuan He, Xinggang Luo, Zhongliang Zhang, Yang Yu

**Affiliations:** 1 School of Information Science and Engineering, Northeastern University, Shenyang 110004, China; hejiahuan0211@163.com (J.H.); yuyang@ise.neu.edu.cn (Y.Y.); 2 School of Management, Hangzhou Dianzi University, Hangzhou 310018, China; zlzhang@hdu.edu.cn

**Keywords:** elderly care services, evolutionary game theory, evolutionary stable strategy, strategy analysis

## Abstract

Population aging poses challenges to the immature elderly care service system in many countries. The strategic behaviors of different participants in the provision of elderly care services in a long-term and dynamic situation have not been well studied. In this paper, an evolutionary game model is developed to analyze the strategic behaviors of two types of participants—the government sectors and the private sectors in provision of elderly care services. Firstly, eight scenarios are analyzed, and the evolutionary process and stable strategies are identified. Then, the behavioral strategies of the two types of participants under demand disturbance and dynamic subsidy strategy are analyzed. Simulation experiments are conducted to explore the influence of different initial conditions and parameter changes on the evolutionary process and results. The obtained observations are not only conducive to a systematic understanding of the long-term dynamic provision of elderly care services but also to the policymaking of the government.

## 1. Introduction

Data from the United Nations show that there are 702.9 million individuals aged over 65 worldwide in 2019, which is expected to increase to 1.55 billion by 2050 [1]. Population aging has posed challenges to many countries around the world as a result of the decline in fertility and mortality in recent years [2]. For example, a heavy burden of disease, the increasing risk of disability, a shortage in the supply of services, and the huge costs for providing elderly care services [3]. Therefore, it is difficult to meet the demand for elderly care services relying only on the provision of government departments. Facing the pressures of population aging, many countries have incorporated private elderly care service providers into the elderly care service system. In developed counties, such as Finland, contracting out to private sectors has been popular since the early 2000s [4]. Moreover, relevant data show that private elderly care service institutions in developing countries have also shown a development trend [5]. In Tianjin, China, there were only four facilities run by the government in the 1980s, but 157 by 2010 (20 were run by the government, and 137 were run by private providers) [5]. The government sectors need to pay attention to the formulation of reasonable policies to attract private sectors participating in the provision of elderly care services, so as to alleviate the supply shortage of elderly care services and promote the development of elderly care service system [2].

The elderly people often suffer from chronic diseases, disability, or semi-disability [6]. Imperfect service management system, poor executive ability, unprofessional service staff, and potential safety hazards may lead to low quality elderly care services, thus increasing the health risks of the elderly. Therefore, the demands of better access and higher quality care services for the elderly cannot be ignored [7]. In view of the profit-seeking characteristics of private service providers and the particularity of the receivers of elderly care services, the government needs to reasonably formulate regulatory policies to ensure the healthy development of the elderly care service system. Meanwhile, the regulatory decisions of the government are also affected by many factors, such as subsidy strategy, punishment strategy, and environmental factors. Therefore, it is worth studying when the government sectors are willing to implement supervision strategy towards the private sectors. The provided services of the private elderly service sectors need to be improved and supervised compared with public elderly service sectors. A series of monitoring data released by the Shanghai Civil Affairs Bureau shows that the service quality of public elderly care institutions in Shanghai is better than that of private elderly care institutions. Meanwhile, those rated as “general quality” and “poor quality” accounted for 18.02% and 1.18%, respectively [8]. For the private service sectors, it is worth studying when the private service sectors choose to provide high-quality or low-quality services. Therefore, what strategies the government sectors should adopt to encourage the private sectors to provide high-quality services are also worthy of being studied.

The issues of elderly care services have attracted attention from many scholars. Existing studies of elderly care services are mainly focused on qualitative analysis of policies and current situations of elderly care services [9,10], empirical studies on the elderly care service system [11], and quantitative research of elderly care services provision strategy [12,13]. It is generally acknowledged that, the provision of elderly care services is an important part in constructing an elderly care service system, while the cooperation between stakeholders and actors is a key factor to a successful system [9]. Therefore, in this paper we try to analyze the behavioral strategies of the government sectors and the private sectors in the provision process of elderly care service system under a quantitative decision framework.

At present, many scholars have studied the optimal provision strategy of elderly care services in a one-time transaction [12,13,14]. However, the following factors are not considered in their studies: (1) The government sectors’ policies may change with time. For instance, Italy has adopted different policies on long-term care at different periods: at the beginning of 21st century, the government adopted cost control measures; subsequently, Italy’s long-term care policies have improved both in terms of the supply of public funds and the quality of services [15]. Moreover, when the government sectors’ policies change, the private sectors may adjust their strategies accordingly. The payoffs of the two types of participants (government sectors and private sectors) in long-term are changing over time and the strategies that could gain higher payoffs will displace the strategies with lower payoffs [16]. Therefore, the decision-making of the participants goes through a dynamic and long-term process and it is difficult for the participants to identify and achieve the optimal strategy in a single game process [17]. (2) In the long-term provision of elderly care services, the strategic choices of government sectors or private sectors have mutual influence on each other and they may not take the best strategy at the beginning of the decision process [18]. When faced with complex situations, the two types of participants may have limited abilities in rational decision-making due to information asymmetry and complex decision-making environments [18]. The two types of participants constantly adjust their strategies through imitation, learning and interaction, and reach a steady state eventually [19]. (3) Changes in population structure, government policy orientation and other factors may have impacts on the demand for elderly care services. Therefore, customer demand for elderly care services may also change over a long period of time. The abovementioned factors may lead to new changes in the elderly care service system, and finally affect the policies of the government sectors and the quality decisions of the private sectors. To solve the abovementioned problems, evolutionary game theory (EGT) is introduced in this research to model the repeated strategic interaction [19] and to study the behavioral decisions of the government sectors and private sectors under bounded rationality and information asymmetry. Through the analysis above, we think that EGT is suitable for analyzing the provision process of elderly care services.

This research aims to study the decision-making process of government sectors and private sectors in the provision of elderly care services in a long-term and dynamic environment. We establish an evolutionary game model to analyze the provision decision of elderly care services with considering that the participants are not completely rational in the long-term decision-making process. Through the analysis of evolutionary game model, we obtain the evolutionary equilibriums of the system under different scenarios, the impacts of different parameters on the evolutionary process and results, and the impacts on the elderly care provision system when facing the changes in environment and policy-making. By systematically analyzing the decision-making process under different scenarios and changes, we obtain relevant management implications to help the policy making of government sectors in elderly care service system. As far as we know, there is no existing research analyzing the decision-making of the government sectors and private sectors in the provision of elderly care services from such a perspective. This research fills this gap and tries to answer the following research questions:

(1) In the long-term and dynamic provision process of elderly care services, the government sectors can adjust the supervision intensity of the private sectors, and the private sectors can set the level of services provided. What supervision strategies do the government sectors adopt under different conditions? Under what conditions are the private sectors willing to deliver high-quality services?

(2) What effect will it have on the decisions of the government sectors and the private sectors if there are the following changes in the long-term provision process of elderly care services:

- If the subsidy, fine, or supervision cost of government sectors is adjusted, or the cost of private sectors is changed, how will the decisions of the private sectors be affected?
- If there is disturbance in service demand within a long period of time, how will the decisions of the two types of participants be affected? Moreover, if the government sectors adopt a dynamic subsidy policy, how will it affect the decisions of the participants?

(3) What policies and measures should the government sectors take to encourage the private sectors to provide high-quality services for the elderly?

The rest of this paper is structured as follows. In Section 2, the relevant literature on elderly care services and the application of EGT are reviewed. The hypothesis and the mathematical model to formulate the behavioral strategies of the participants are presented in Section 3. The demand disturbance on the evolutionary process and results are presented in Section 4. In Section 5, an in-depth analysis of the game under dynamic subsidy policy is shown. Finally, discussions and brief conclusions are given in Section 6 and 7. Some of the theoretical proofs and numerical simulations are listed in the Appendix A, Appendix B, Appendix C and Appendix D.

## 2. Literature Review

This research mainly relates to two literature streams. From the perspective of the research problem, it is related to elderly care services and its provision. On the other hand, from the modeling perspective, it is related to the EGT and its application.

### 2.1. Elderly Care Services and Its Provision

Problems and challenges faced in the provision process of elderly care services have raised the attention of many researchers. Take China as an example, the involvement of private sectors in the provision of elderly care services is increasing while the shortage of long-term care staff and the poor quality of services are worrying. The shortage of care beds is also an urgent problem; China has about half as many long-term care beds per 1000 elderly people as most developed countries do [20]. Moreover, the funding for the public long-term care is limited [21]. The weak regulatory framework and the lack of enforcement capacity are also challenges that the government policymakers face in regulating the rapidly growing elderly care sectors [9]. Therefore, for the government sectors, providing high quality services under limited budget is a problem that needs to be solved. In the study of elderly care services provision, the optimal policy of service provision is one of the concerns of scholars. For instance, Pestieau and Sato [12] analyzed the optimal policy of the mixed provision of formal and informal care to dependent parents under complete information. Their study also identified the right mix of long-term care under redistributive situations. On the other hand, Kuhn and Nuscheler [13] showed the optimal policy with and without the involvement of nursing homes under asymmetric information. Specifically, they established a theoretical model to analyze the long-term care provision under adverse selection and found that the provision of formal care is an effective way to solve the supply problem of elderly care services. Moreover, some scholars focused on the quality and price competition in long-term care [22]. The above studies focused on analyzing the optimal supply strategy and competition in the long-term care service provision. However, the above studies were all under a single scenario. They mainly focused on optimal strategies but ignored the evolutionary results under different scenarios and the evolution process of the system in achieving the evolutionary stable strategies, which are also important for the provision process analysis of elderly care services. They did not take the impacts that government sectors might have on the provision process of elderly care services into consideration either. Besides these quantitative analyses, some researchers focused on the supply policy of elderly care services. For example, Feng et al. [2] analyzed the policy challenges that China faces in establishing a long-term care system for its rapidly growing age population. Gori et al. [15] explored the impact of cost control strategies on long-term care provision. The research also showed that the Italian government has adopted different policies at different times. Some empirical studies were also conducted on elderly care services. Chang et al. [23] adopted a cross-sectional study of the relationship between privately or publicly owned elderly care facilities and their service quality. Taking Nanjing and Tianjin cities in China as examples, they identified a shortage of manpower in the private sector and that selective admission policies are better for healthier people in the public sector. Tynkkynen et al. [4] analyzed contracting out elderly care and primary healthcare services through empirical research. They found that contracting with the private sector can improve performance, service quality, and also be good for the local economy. Goharinezhad et al. [9] studied the challenges of elderly care in developing countries. Specifically, they used qualitative analysis to study the current situation of elderly care in Iran. Five challenges, including policymaking and health-based care services, were identified in their study. Some scholars focused on the privatization of social services. For example, Stolt et al. [24] analyzed the privatization of elderly care services in Sweden based on statistical analysis to compare service quality between private and public elderly care services. In addition, the factors affecting for-profit supplier entry into the long-term care market under price regulation were studied by Mutsumi et al. [25]. The above qualitative or empirical analysis studied the provision of elderly care services from different perspectives such as policy analysis, privatization of elderly care services and the problems faced by the provision of elderly care services. However, they all ignored the analysis on the interaction within and between the two types of participants.

### 2.2. Evolutionary Game Theory and Its Application

Game theoretic approaches can provide a quantitative decision framework for modeling, analyzing as well as predicting the behaviors of the players in the game [17]. As is well-known, orthodox game theory assumes that players in a game are fully rational and know the rules of the game well, hence they can make the optimal decision quickly [26]. However, when it comes to the decision-making on more complex social issues, these assumptions are often inconsistent with the actual situations, as players often show bounded rationality and it is impossible for each player to be acquainted with the complete information of others. Moreover, they may even make wrong decisions and the strategies they made may affect each other [27]. The provision of elderly care services is a complex and long-term social problem. During the service provision process, the strategies of the government sectors and the private sectors may be affected by many factors, such as cost variations, environmental factors, and the effects on each other. The existence of information asymmetry makes the government sectors and the private sectors unable to make the optimal decision quickly and accurately. Hence, static analysis is unsuitable for analyzing the dynamic and complex process of elderly care services provision. EGT provides a way of illustrating dynamics. It supposes that the players are with bounded rationality and the players making their decisions under imperfect information [26]. The players interact with the others for multiple rounds by adopting different strategies; at the same time, the state of the interaction will be changed overtime according to the replication games [28]. Strategies that receive higher profits will win over the ones with lower profits and then spread in the population [16]. EGT mainly concentrates on the interaction among different participants and focuses on the dynamic process and results of strategy change [29]. Therefore, compared with some traditional game methods, EGT is more suitable for our research to analyze the long-term provision process of elderly care services.

Recently, EGT has attracted attention from researchers for solving economic and social problems [30]. For instance, some scholars used EGT to analyze the decision processes in public–private partnerships (PPP). Xue et al. [31] proposed a new private capital investment method and focused on the impacts of private capital investment on public transportation. They used an evolutionary game model as a quantitative analysis tool for administrators and the results showed that investment strategy is an evolutionary stable strategy (ESS). Gao and Zhao [32] established an evolutionary game model to study the relationships between the government and investors in energy power projects. Some policy suggestions were also given from the government perspective. Li et al. [33] analyzed the evolutionary regularity between the government sectors and the private sectors, and suggestions were proposed to restrain the private and improve the supervision efficiency of the public sector. EGT has also been widely used in supply chain strategy selection. For instance, Xiao and Chen [34] developed a three-stage evolutionary game model of the supply chain. They aimed to study the strategy selection of retailers based on wholesale price. Further, Xiao and Yu [26] used an indirect evolutionary game model to study the evolutionary stability of the retailers. They also considered demand disruption and raw material supply disruption to determine their impacts on the ESS of retailers. EGT is also an effective tool to analyze cooperation strategies. Ji et al. [30] developed an evolutionary game model to discuss the cooperation strategy selection of suppliers and manufactures, and the evolutionary stability of participants was also studied through the replicator dynamic system. Cheng and Gong [35] used EGT to study the technology-licensing cooperation between two firms under two licensing schemes. They also analyzed the influence of parameters on the cooperative probability under different licensing schemes. Hu et al. built up an evolutionary game model to analyze the interest-related decision-making behaviors of the upstream and downstream stakeholders [36]. The above studies all showed that EGT is an effective way to analyze the complex and dynamic social problems.

From the above-cited studies, we can see that existing studies on elderly care services mainly focused on policy research, qualitative analysis, and empirical analysis. Although there are some papers on quantitative analysis of the provision of elderly care services, they all focused on the optimal provision strategies, but ignored the impacts of the change in participants’ behavior strategies, the interaction between participants, as well as the change of environmental and other related factors to the elderly care services provision process. Based on the analysis above, we believe that the EGT is more suitable for us to analyze the long-term provision process of elderly care services. To the best of our knowledge, there is no research using EGT to analyze the behavior strategies of the government and the private sector in the provision of elderly care services. As such, we develop an evolutionary game model to analyze the behavior strategies of government sectors and private sectors in the provision process of elderly care services under different situations.

## 3. Model

In the following, we take China as an example to describe the decision problem. The government has built up a dual system of institutional elder care: one is managed and owned by the government; the other is market driven and run by the private sectors [2]. The government’s role is shifted from the direct “provider” to “purchaser and regulator”. By following the strategic shift, a series of policies and strategies are carried out, and the private sectors operate elderly care service institutions with government support and subsidies (e.g., tax exemptions, subsidies for beds) for construction and operation [2]. For instance, in Nanjing, China, the government sector provides a CNY 2000–4000 subsidy to the suppliers per bed [4]. Moreover, Xi’an, China, has introduced two insurance systems, one for accidental injuries to the elderly and the other for the comprehensive liability of elderly care institutions [37]. The government has implemented many policies and measures to promote the development of elderly care service system, but there are still many problems in service quality of elderly care institutions. For instance, in 2017, more than 30,000 quality problems in elderly care institutions were found in Beijing [38]. The goal of private sectors is profit maximization; to ensure the service quality of elderly care service institutions, government regulation is an essential way to promote the comprehensive development of the elderly care service system. China has introduced minimum standards for the management of elderly care services providers since 2001. The standards stipulate the basic service standards, facility operations, relevant legal liability, and penalties for service providers that do not meet standards. For instance, Beijing, China, had implemented measures for the management of operating subsidies for elderly care institutions since 2018. Subsidies for nursing homes will be removed when they provide low-quality elderly care services [39]. In recent years, the government policies on elderly care services have developed from simplicity to diversification [40]. At the same time, the provision of elderly care services shows the characteristics of dynamic and long-term. The demographic structure and policy changes have caused changes in the demand for elderly care services [41], and private elderly care service institutions have shown an increasing trend [5].

Based on the analysis of the actual situations, we formulate the behaviors of the government sectors and the private sectors in the provision of elderly care services as an evolutionary game. The model is described in detail as follows.

### 3.1. Model Hypotheses and Description

To facilitate modeling, we present the following assumptions:

(1) There are two types of participants in the provision process of elderly care services—the government sectors and the private sectors. Similar settings can be found in Li et al. (2016) [33].

(2) Each of the two types of participants has two strategies to choose from. The government sectors have two types of choices: implement supervision and implement no supervision. It costs S if a government sector implements supervision strategy. The private sectors choose to provide high-quality services or provide low-quality services.

(3) Both of the two types of participants are with bounded rationality. Because of incomplete information, they have limited abilities in rational cognition and decision-making. However, each entity of a type of participants may interact with each other and can learn constantly to adjust its strategy [29].

(4) Provision of high-quality elderly care services brings a positive reputation benefit to the government sectors, which we defined as R. Moreover, when the government sectors implement supervision while the private sectors serve high-quality services, it brings an additional positive utility K to the government sectors. Similar settings can be found in Wang et al. (2020) [42].

(5) Many elderly people are suffering from long-term chronic diseases; they are at higher risks of sudden illness and accidents. Hence, we assume that the private sectors providing low-quality services face a risk cost, V.

For the government sectors, it is necessary to formulate reasonable policy on the provision of elderly care services and regulate the behavior of private sectors. Moreover, elderly care services have the characteristics of high investment costs, long return cycles, and low profit, hence the government sectors need to make supporting policies for the private sectors while regulating them. The private sectors are the direct providers of elderly care services. It is unrealistic to achieve high-quality service provision only by the self-discipline of the private sectors without supervision of the government. Therefore, the supervision strategy should be the dominating strategy of the government sectors, and for the provision of elderly care services, supervision strategy, and high-quality services are ideal decision-making results. The private sectors can get a per capita subsidy from the government sectors. However, when the private sectors provide low-quality services while the government sectors implement supervision, the private sectors will be punished by the government and receive no subsidy, the government will compensate the customers in the meanwhile. The two types of participants pursue social value or economic value in the provision process of elderly care services. For the sake of analysis, we express it as fitness here.

The main notations of the model and their definitions are listed in Table 1.

### 3.2. Establishment of the Model

The game players have different strategies and payoffs. The pure strategies of the government sectors are: (1) Is: the government sectors implement supervision; (2) Ns: the government sectors implement non-supervision. The pure strategies of the private sectors are: (1) Hs: the private sectors provide high-quality services; (2) Ls: the private sectors provide low-quality services. According to the assumptions above, when the government sectors choose Is and the private sectors choose Hs, the government sectors obtain positive revenue from the high-quality services (*R*) and its supervision (*K*). Moreover, the government sectors have to pay for the supervision cost (*S*) and the subsidy (θq) to the private sectors. Then, the total revenue of the government sectors is the difference between the positive revenue and the costs, corresponding to R−S−θq+K in Table 2. For the private sectors, the total revenue is the difference between the positive revenue (benefits from patients (Pq) and subsidies from the government (θq)) and the service costs ($C_{0}q$), corresponding to $Pq-C_{0}q+\theta q$ in Table 2. When the government sectors choose Is and the private sectors choose Ls, the government sectors collect fines (*F*) from the private sectors, while pay for the supervision cost (*S*) and the subsidies to the customers (*M*) who receive low-quality services, corresponding to −S+F−M in Table 2. For the private sectors, the total revenue is the difference between the positive revenue (benefits from the customers) and the costs (service cost, the fines for low-quality services and the risk of low-quality services), corresponding to $Pq-C_{1}q-F-V$ in Table 2. Similarly, we can get the rest of the payoff matrix based on the assumptions. Table 2 depicts the payoff matrix of government sectors and private sectors in the provision of elderly care services.

We thus obtain the expected return of the government sectors. If the government sectors choose to implement supervision (non-supervision), the payoff function is $E_{g1}$ ($E_{g2}$). Then, the average expected revenue of the government sectors is $\bar{E_{g}}$:

(1) $$E_{g1}=y(R-S-\theta q+K)+(1-y)(-S+F-M)$$

(2) $$E_{g2}=y(R-\theta q)+(1-y)(-\theta q)$$

(3) $$\bar{E_{g}}=xE_{g1}+(1-x)E_{g2}$$

The replicator dynamic equation can be used to describe the evolution of participants’ strategy over time. In a replicator dynamic system, the growth rate of a strategy is equal to the difference between its fitness and population average fitness [43]. Therefore, the replicator dynamic equation of the government sectors when implementing supervision can be formulated as follows:

(4) $$F(x)=\overset{\cdot }{x}=\frac{dx}{dt}=x(E_{g1}-\bar{E_{g}})=x(1-x)\left(y(K-F+M-\theta q)+F-S-M+\theta q\right)$$

Similarly, we obtain the replicator dynamic equation of the private sectors providing high-quality services:

(5) $$F(y)=\overset{\cdot }{y}=\frac{dy}{dt}=y(E_{e1}-\bar{E_{e}})=y(1-y)\left(x(F+\theta q)-C_{0}q+C_{1}q+V\right)$$

Equations (4) and (5) constitute the replicator dynamic system for the provision of elderly care services.

To simplify the expressions, we define the following four expressions, $\pi_{1}=F+\theta q-C_{0}q+C_{1}q+V$, $\pi_{2}=-C_{0}q+C_{1}q+V$, $\pi_{3}=K-S$, and $\pi_{4}=\theta q+F-S-M$, where $\pi_{1}$ represents that, relative to providing low-quality services, the net revenue of the private sectors for providing high-quality elderly care services when the government sectors implement supervision strategy (we call it the net revenue for providing high-quality elderly care services for short); $\pi_{2}$ represents that, relative to providing low-quality services, the net revenue of the private sectors for providing high-quality elderly care services when the government sectors implement non-supervision strategy; $\pi_{3}$ represents that, relative to non-supervision, the net revenue of the government sectors for implementing supervision when the private sectors provide high-quality elderly care services (we call it the net revenue of the government sectors implementing supervision for short); and $\pi_{4}$ represents that, relative to non-supervision, the net revenue of the government sectors for implementing supervision when the private sectors provide low-quality elderly care services. The revenue of providing low-quality services is greater than that of providing high-quality services once when the government implements non-supervision, hence we always have $\pi_{2}<0$.

### 3.3. Equilibrium Analysis

From the above replicator dynamic system, we obtain the following proposition.

**Proposition** **1.** *For the replicator dynamic system of elderly care services provision given by Equations (4) and (5), we have:*

*(i) Fixed points (0, 0), (1, 0), (0, 1), and (1, 1) are the equilibriums of the system.*

(ii) $\left(x^{*},y^{*}\right)$ is also an equilibrium of the replicator dynamic system; if $\pi_{1}>0$, $\pi_{2}<0$, $\pi_{3}>0$ and $\pi_{4}<0$, or $\pi_{1}>0$, $\pi_{2}<0$, $\pi_{3}<0$ and $\pi_{4}>0$, where $x^{*}=\frac{C_{0}q-C_{1}q-V}{F+\theta q}$, $y^{*}=\frac{S+M-F-\theta q}{K+M-F-\theta q}$.

See the Appendix B for the proof. (Other proofs of the article are also given in Appendix B)

The equilibrium depends on the ratio of participants’ initial strategies and the sign symbol of the differential equation in corresponding interval [44]. According to the linearization method given by Hofbauer and Sigmund [45], we can use the Jacobian matrix to evaluate the stability of the equilibrium strategy. If conditions DetJ>0 and TrJ<0 are satisfied, the equilibrium point is an ESS [19]. We express matrix J of the replicator dynamic system as follows:

(6) $$J=\left[\begin{array}{l} \partial F(x)/\partial x\;\partial F(x)/\partial y\\ \partial F(y)/\partial x\;\partial F(y)/\partial y \end{array}\right]=\left[\begin{array}{l} \left(1-2x)\left[y(K-F+M-\theta q)+F-S-M+\theta q\right]\;x(1-x)(K-F+M-\theta q\right)\\ y(1-y)(F+\theta q)\;(1-2y)(x(F+\theta q)-C_{0}q+C_{1}q+V) \end{array}\right]$$

Then, DetJ and TrJ of matrix J are:

(7) $$\begin{array}{r} DetJ=(1-2x)[y(K-F+M-\theta q)+F-S-M+\theta q](1-2y)[x(F+\theta q)-C_{0}q+C_{1}q+V]-\\ x(1-x)(K-F+M-\theta q)y(1-y)(F+\theta q) \end{array}$$

(8) $$TrJ=(1-2x)[y(K-F+M-\theta q)+F-S-M+\theta q]+(1-2y)[x(F+\theta q)-C_{0}q+C_{1}q+V]$$

The expressions of DetJ and TrJ at the four fixed evolutionary equilibriums are shown in Table 3.

We get eight scenarios according to the sign symbol of $\pi_{1\sim 4}$. After dividing the game into eight scenarios, we obtain the signs of DetJ and TrJ at the evolutionary equilibriums of each scenario. According to the method of Friedman [19], we obtain the local stability of the evolutionary equilibriums, as shown in Table 4. Considering the space limitation, we mainly introduce the first four scenarios below, and put the last four scenarios in Appendix A.

**Figure 1:**
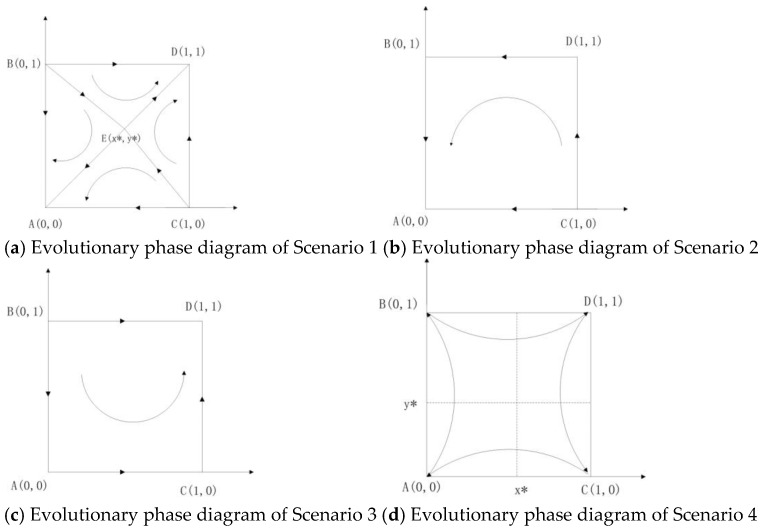
Evolutionary phase diagrams of Scenario 1 to 4.

**Scenario** **1.** *When $\pi_{1}>0$, $\pi_{2}<0$, $\pi_{3}>0$, $\pi_{4}<0$ are satisfied, the evolutionary stable strategy of the game is (1, 1) and (0, 0). The evolutionary phase diagram of scenario 1 is shown in Figure 1. In this case, the additional revenue K of the government sectors is greater than the supervision cost; the sum of subsidies and penalties is less than the sum of supervision cost and customers’ compensation. Moreover, for the private sectors, the total cost of providing high-quality services is lower than the sum of the penalties, subsidies, costs, and risk value of providing low-quality services. The evolutionary result depends on the initial state of the system. When the initial state is in the region ABEC in Figure 1a, the evolutionary equilibrium of the system is (0, 0). The government sectors implement non-supervision and the private sectors provide low-quality services. When the initial state is in the region BECD, the evolutionary equilibrium of the system is (1, 1). The government sectors implement supervision and the private sectors provide high-quality services. The larger the area of the region, the more likely it is to evolve towards the corresponding evolutionary result.
*

**Scenario** **2.** *When $\pi_{1}>0$, $\pi_{2}<0$, $\pi_{3}<0$, $\pi_{4}<0$ are satisfied, the evolutionary stable strategy of the game is (0, 0). In this case, regardless of the service quality provided by the private sectors, the net benefit of the government sectors implementing supervision is negative. Therefore, the government sectors will implement non-supervision. Under the negative supervision policy, the private sectors will choose to provide low-quality services. The evolutionary result is that the government sectors implement non-supervision and the private sectors provide low-quality services.*

**Scenario** **3.** *When $\pi_{1}>0$, $\pi_{2}<0$, $\pi_{3}>0$, $\pi_{4}>0$ are satisfied, the evolutionary stable strategy of the game is (1, 1). In this case, the net benefit of the government sectors implementing supervision is positive, regardless of the service quality provided by the private sectors. The additional revenue K of the government sectors is greater than the supervision cost, the sum of subsidies and penalties is greater than the sum of supervision cost and customers’ compensation. For the private sectors, the net benefits for providing high-quality services are positive, which means that the total cost of providing high-quality services is lower than the sum of the penalties, subsidies, costs and risk value of providing low-quality services. Then, the government sectors will implement supervision and the private sectors will provide high-quality services eventually.*

**Scenario** **4.** *When $\pi_{1}>0$, $\pi_{2}<0$, $\pi_{3}<0$, $\pi_{4}>0$ are satisfied, there is no stable equilibrium point. The system will not reach a stable state and no certain strategy will be reached.*

The phase diagrams of the first four scenarios are shown in Figure 1.

According to the evolutionary equilibrium results of the eight scenarios, the following proposition can be obtained.

**Proposition** **2.**

*(i)*  If $\pi_{1}>0$ and $\pi_{3}>0$, the equilibrium point (1, 1) is an ESS.
*(ii)*  *If $\pi_{4}<0$, the equilibrium point (0, 0) is an ESS.*
*(iii)*  *If $\pi_{1}<0$ and $\pi_{4}>0$, the equilibrium point (1, 0) is an ESS.*

**Figure 2:**
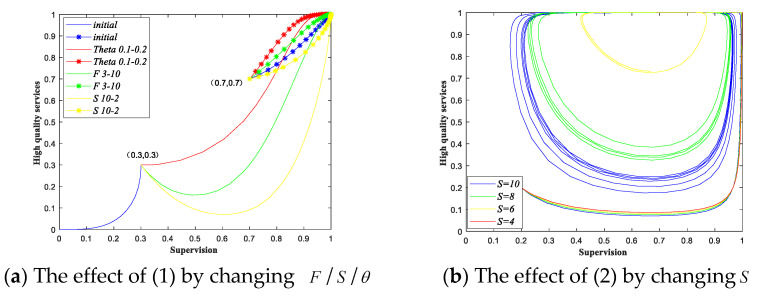
Effect of parameter variation on the evolutionary results under different initial conditions.

Based on the analysis of each scenario above, we can easily get Proposition 2, where (i) corresponds to scenario 1 and 3; (ii) corresponds to scenario 1, 2, 5 and 6; (iii) corresponds to scenario 7 and 8. For (i), when conditions $\pi_{1}>0$ and $\pi_{3}>0$ are met, the game will eventually evolve to {Is, Hs} in scenario 3 or when the initial state in scenario 1 is in region ABEC in Figure 1a. As we have discussed above, supervision-high quality services are ideal decision for the provision of elderly care services. For the government sectors, if the conditions in Proposition 2(i) are not met, the values of $\pi_{1}$ and $\pi_{3}$ can be adjusted to promote the game evolving to the ideal state. The values of parameters F, θ and S will affect the value of $\pi_{1}$/$\pi_{3}$. The government sectors can impose a high penalty/unit subsidy strategy or reduce the cost of supervision, so that the value of $\pi_{1}$/$\pi_{3}$ will increase, and when the conditions $\pi_{1}>0$ and $\pi_{3}>0$ are met, the system can get the ideal strategy result. From the economic perspective, hefty fines for poor quality services of the private sectors increase the cost of providing low-quality services; increasing the subsidy for unit service will increase the revenue of private sectors. The private sectors will choose to provide high-quality services as long as the net revenue of providing high-quality services is positive. Hence, the increasing in F and θ will have positive impact on the private sectors for providing high-quality services. For the government sectors, improving work efficiency to reduce its supervision cost is helpful for the government sectors to evolve towards providing supervision. Under supervision, the private sectors may choose to provide high-quality services, otherwise the private sectors will provide poor quality services to save costs. For (ii) and (iii), the private sectors will eventually provide low-quality services when the government sectors implement non-supervision. Moreover, even if the government sectors eventually evolve to implement supervision, the private sectors will evolve to provide low-quality services if the net revenue of providing high-quality services is negative. To have a better understanding of the above conclusions, we show a set of numerical simulations. Parameters in this paper are derived from the actual data of private elderly care service institutions in some cities in China, as well as the relevant policies and regulations of elderly care services. See Appendix C for the source and range of parameter values. We set the initial parameters as follows in Table 5. We can easily obtain the conclusions from the examples and Figure 2 below.

The parameters in the first row of Table 5 correspond to scenario 1. We give two initial strategy states ($x_{0},y_{0}$). Taking the initial strategy state (0.3, 0.3) as an example, we can see the evolutionary result is (non-supervision, low quality services). As the value of F and θ go up, or the value of S goes down, until the essential conditions are met, then the evolutionary result of the system is (supervision, high quality services). The parameters in the second row of Table 5 correspond to scenario 4. Taking the initial strategy state as (0.2, 0.2), we can see that the evolutionary paths of the two types of participants cannot reach a stable state. In scenario 4, $\pi_{3}<0$, according to the analysis above we can adjust the value of the supervision cost. As the supervision cost decreases, the evolutionary path of the system is still in a state of fluctuation, but it has a tendency to evolve towards the ideal direction. Finally, when the condition $\pi_{3}>0$ is met, the evolutionary strategy of the system is (supervision, high-quality services). Similar numerical simulations on other scenarios are done, which are presented in the Appendix D.

The analysis above mainly focuses on the impact of policy changes of the government sectors on evolutionary result. For the private sectors, the cost difference between different quality services affects the value of $\pi_{1}$. Therefore, the evolutionary results of the system will be affected. The following conclusions can be drawn from the above analysis.

**Observation** **1.** *When $\pi_{1}>0$ is met, that is when the cost difference between different quality services provided by the private sectors satisfies $C_{0}-C_{1}<\frac{F+\theta q+V}{q}$ and when $\pi_{3}>0$ is met, the game will eventually evolves to (1, 1) in scenario 3, or to (1, 1) or (0, 0), depending on the initial state and parameter values in scenario 1.*

Observation 1 shows that, it is difficult to achieve an ideal evolutionary result when the cost difference between different quality services is too large. Moreover, which evolutionary equilibrium the game eventually evolves to depends on the initial states of the participants and parameter values. We can make the game eventually evolve to (1, 1) by adjusting the related parameters.

### 3.4. The Impact of Different Factors on ESS

In scenario 1, the evolutionary result can be either (supervision, high-quality services) or (non-supervision, low quality services). The probability of the evolutionary result in which direction depends on the area of ABEC and BECD in Figure 1a. Based on the coordinates of point $E(x^{*},y^{*})$, we can obtain the area of BECD shown in Equation (9). The larger the area of region BECD is, the more likely the evolutionary result is (supervision, high quality services). Therefore, we need to increase the area of region BECD. The influences of different factors on the evolutionary results are as follows:

(9) $$S_{BECD}=\frac{1}{2}\left(1-\frac{C_{0}q-C_{1}q-V}{F+\theta q}\right)+\frac{1}{2}(1-\frac{S+M-F-\theta q}{K-F+M-\theta q})$$

**Proposition** **3.** *In Scenario 1, the probability of the game evolving to (1, 1) increases when the value of $C_{1}$ (F, θ) increases or the value of $C_{0}$ (q, S, M) decreases.*

From Proposition 3, we can see that, the higher the unit cost of low-quality services is, the higher the fines of providing low-quality services are, and the higher the unit subsidy for providing elderly care services are, the higher the probability for the system evolving to (supervision, high-quality services) is. Moreover, the smaller the unit cost of high-quality services is, the fewer the elderly served by a single private sector is, the smaller the government’s supervision cost is, and the smaller the compensation paid to the customer is, the higher the probability for the system evolving to (supervision, high-quality services) is. For the private sectors, providing low-quality elderly care services may lead to fines and the failure to obtain subsidies from the government sectors. Meanwhile, low-quality services will increase the operation risks. Hence, when the costs difference between different quality services is not enough to make up for the loss of low-quality services, the private sectors will choose to provide high-quality services. In addition, the number of elderly people served by a single service provider should not be too large ($q<\frac{F+\theta q+V}{C_{0}-C_{1}}$), otherwise the service quality will be reduced. For the government sectors, appropriate measures can be taken, such as reducing the supervision cost and raising the fine, so as to promote the game to evolve towards the ideal result.

**Figure 3:**
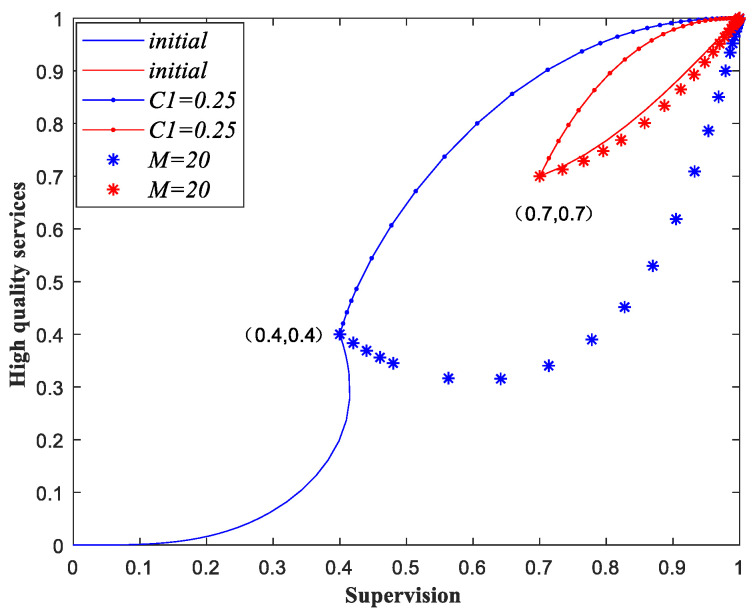
Influence of variation of parameters $C_{1}$ and M on the evolutionary results.

To have a more intuitive understanding of Proposition 3, we provide a numerical simulation below. The initial values of the parameters are the same as those in (1) of Table 5. By changing the different parameters, we take $C_{1}$ and M as examples and obtain the evolutionary paths in Figure 3.

The evolutionary paths of two initial strategy states (0.4, 0.4) and (0.7, 0.7) are shown in Figure 3. We can see that when the parameters remain unchanged, the evolutionary results of the two initial strategy states are (0, 0) and (1, 1), respectively. When the cost of providing low-quality services increases or the compensation to the customers decreases, both the evolutionary results of the two initial states are (1, 1). Hence, when the government sectors raise the penalty and unit subsidy, reduce compensation, or improves supervision efficiency, the probability of the game evolving to (1, 1) increases. Moreover, if the cost differentials between different quality services decreases, the probability of the game evolving to (1, 1) increases. Moreover, the demand of service can also influence the final evolutionary result.

## 4. Evolutionary Stable Strategies under Demand Disturbance

The fluctuation in the demand for elderly care services will not be too large in the short term. However, in the long run, government policy, changes in demographic structure, regional disparity, or some haphazard events may affect the demand for elderly care services. Let Δq represent the change in demand and q+Δq represents the demand for elderly care services under disturbance.

Since the evolutionary result of scenario 3 is ideal from the government’s perspective, we take scenario 3 as an example to explore the impacts of changes in demand on the evolutionary results.

When the conditions of scenario 3 are met, the following statements hold: $0<C_{0}q-C_{1}q-V<F+\theta q$ and S+M−F−θq<K+M−F−θq<0. Therefore, when the change in demand Δq satisfies $\max\left(\frac{V}{C_{0}-C_{1}},\frac{S+M-F}{\theta }\right)-q<\Delta q<\frac{F+V}{C_{0}-C_{1}-\theta }-q$, the eventual equilibriums have the same local stability as those without demand disturbance, and equilibrium point (1, 1) is the ESS. The question is what impacts it will have on the final evolutionary equilibrium when the change in demand is not in the range above. We have already assumed that the revenue from providing low-quality services is greater than the revenue from providing high-quality services when the government implements non-supervision strategy. That is, the change in demand must satisfy condition $\Delta q>\frac{V}{C_{0}-C_{1}}-q$. When the other settings of scenario 3 remain unchanged, we have the following proposition.

**Proposition** **4.** *When considering the out-of-range demand disruption, we have:*

*(i) Equilibrium point (1, 0) is the ESS when $\frac{S+M-F}{\theta }<\frac{V}{C_{0}-C_{1}}$ and the change in demand Δq satisfies $\Delta q>\frac{F+V}{C_{0}-C_{1}-\theta }-q$.*

*(ii) Equilibrium point (0, 0) and (1, 1) are the ESS when $\frac{V}{C_{0}-C_{1}}<\frac{S+M-F}{\theta }<\frac{F+V}{C_{0}-C_{1}-\theta }$ and $\frac{V}{C_{0}-C_{1}}-q<\Delta q<\frac{S+M-F}{\theta }-q$.*

*(iii) Equilibrium point (1, 0) is the ESS when $\frac{V}{C_{0}-C_{1}}<\frac{S+M-F}{\theta }<\frac{F+V}{C_{0}-C_{1}-\theta }$ and $\Delta q>\frac{F+V}{C_{0}-C_{1}-\theta }-q$.*

**Figure 4:**
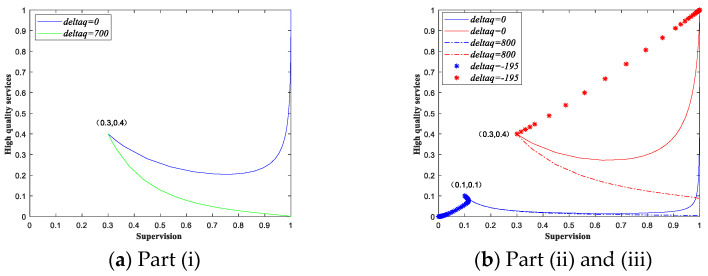
Evolutionary paths of Scenario 3 under demand disturbance.

There are three main nodal values $\frac{S+M-F}{\theta }$, $\frac{V}{C_{0}-C_{1}}$ and $\frac{F+V}{C_{0}-C_{1}-\theta }$ used to analyze the impacts on the final evolutionary results when the change in demand Δq is not in the range $\max\left(\frac{V}{C_{0}-C_{1}},\frac{S+M-F}{\theta }\right)-q<\Delta q<\frac{F+V}{C_{0}-C_{1}-\theta }-q$. From the above analysis, for scenario 3, we can conclude that, when facing demand disturbance, if the change in demand satisfies $\Delta q>\frac{F+V}{C_{0}-C_{1}-\theta }-q$, the private sectors will select to provide low-quality services eventually under disturbance even if the government sectors adopt supervision strategy. When the change in demand meets condition $\frac{V}{C_{0}-C_{1}}-q<\Delta q<\frac{S+M-F}{\theta }-q$, the evolutionary equilibrium will be arrived at (1, 1) or (0, 0), depending on the initial state and parameter values. As such, the proposition above may be difficult to understand. We provide a numerical simulation to show the evolutionary paths under demand disturbance in Figure 4. For example, the values of the parameters are:F=10, $C_{0}=0.3$, q=300, S=2, M=20, V=30, K=20, $C_{1}=0.1$, θ=0.1 in Figure 4a. The initial strategy state is set as (0.3, 0.4). We can see that the evolutionary result is (1, 1) in the initial state, when the change in demand is in the range in part (i), the game evolves to (1, 0). The private sectors choose to provide low-quality services although the government sectors implement a supervision strategy. In Figure 4b, we set the initial strategy state to be (0.1, 0.1) and (0.3, 0.4), and the values of parameters are F=10, $C_{0}=0.3$, q=300, S=2, M=20, V=10, K=20, $C_{1}=0.18$, θ=0.1. We can see that the evolutionary result is (1, 1) of the two initial states, the figure shows the evolutionary paths of two initial states under two different disturbance levels. When the demand disturbance is 800, the evolutionary results of the two different initial states are both (1, 0). When the demand disturbance is −195, the initial strategy state of (0.1, 0.1) evolves to (0, 0), while the (0.3, 0.4) evolves to (1, 1) quickly than the initial state without demand disturbance. Hence, the part (ii) and (iii) of Proposition 4 is verified and the final evolutionary results are related to the initial states and the parameters of the game. Through a similar analysis process, we can obtain the evolutionary paths and results for the game in other scenarios, we omit the specific process due to space considerations.

## 5. Evolutionary Stable Strategies under Dynamic Subsidy Policy

We consider the scenario where the government sectors change the subsidy policy to a dynamic one. That is, the government sectors provide a high subsidy to the private sectors when the percentage of providing high-quality services is low; otherwise, the government sectors adopt the policy of reducing the subsidy. Assume that the government subsidy is inversely proportional to the proportion of high-quality services. The subsidy then changes from θq to B(1−y), where B represents the maximum government subsidy. We define f(y)=B(1−y) and bring it into the replicator dynamic system represented by Equations (4) and (5), where the replicator dynamic system under dynamic subsidy can be described as follows:

(10) $$F{\left(x\right)}^{\prime }=\frac{dx}{dt}=x(1-x)\left(y(K-F+M-f(y))+F-S-M+f(y)\right)$$

(11) $$F{\left(y\right)}^{\prime }=\frac{dy}{dt}=y(1-y)(x(F+f(y))-C_{0}q+C_{1}q+V)$$

The four fixed equilibriums of the system are $A^{\prime }(0,0)$, $B^{\prime }(0,1)$, $C^{\prime }(1,0)$, and $D^{\prime }(1,1)$, and the point $E^{\prime }(\frac{C_{0}q-C_{1}q-V}{F+B(1-y)},\frac{S+M-F-B(1-y)}{K-F+M-B(1-y)})$ is also an equilibrium if $0<\frac{C_{0}q-C_{1}q-V}{F+B(1-y)}<1$ and $0<\frac{S+M-F-B(1-y)}{K+M-F-B(1-y)}<1$ are satisfied. The Jacobian matrix of the replicator dynamic system is as follows:

(12) $$J^{\prime }=\left[\begin{array}{l} \partial F(x)/\partial x\;\partial F(x)/\partial y\\ \partial F(y)/\partial x\;\partial F(y)/\partial y \end{array}\right]=\left[\begin{array}{cc} (1-2x)\left[y(K-F+M-f(y))+F-S-M+f(y)\right] & x(1-x)(K-F+M-f(y)-yf^{\prime }(y)+f^{\prime }(y))\\ y(1-y)(F+f(y)) & \left(1-2y)(x(F+f(y))-C_{0}q+C_{1}q+V)+xy(1-y)f^{\prime }(y\right) \end{array}\right]$$

The expressions of DetJ and TrJ at the evolutionary equilibriums are given in Table 6.

When $F-C_{0}q+C_{1}q+V>0$, only scenarios 1–4 exist. Moreover, the evolutionary stability results under a dynamic subsidy remain the same as in scenarios 1–3. If condition $F-C_{0}q+C_{1}q+V>0$ is not met, the new replicator dynamic system could not achieve evolutionary stability at (1, 1), which is the ideal state from the government’s perspective.

Therefore, when the government sectors choose to implement a dynamic subsidy strategy, formulating a reasonable penalty policy for the private sectors providing low-quality services is necessary. The evolutionary stable results of scenarios 1–3 under the dynamic subsidy are as same as the replicator dynamic system in Equations (4) and (5). To understand the impacts of the dynamic subsidy policy on government spending, we have the following observation for Scenarios 1 and 3.

**Observation** **2.** *When the conditions of scenario 1 are met, if the subsidy of the government sectors meets condition S+M−F>θq>(1−y)B, the government sectors have a lower subsidy cost for choosing dynamic subsidy strategy. Similarly, when the conditions of scenario 3 are met and the subsidy of the government sectors meet condition S+M−F<(1−y)B<θq, the government sectors can pursue a lower subsidy cost by implementing the dynamic subsidy strategy.*

The observation above is obvious if the subsidy under dynamic subsidy policy B(1−y) is below θq. Since the government sectors are often under the pressure of a limited budget, when the above conditions are met, it is beneficial for the government sectors to implement the dynamic subsidy strategy.

When point $E^{\prime }$ is also an evolutionary equilibrium of the game, we discuss the effects of parameter variation on the evolutionary results. In scenarios 1 and 4, they both have five evolutionary equilibriums $A^{\prime }$, $B^{\prime }$, $C^{\prime }$, $D^{\prime }$ and $E^{\prime }$, and we analyze the evolutionary processes of the two game participants under dynamic subsidy strategy. For equilibrium point $E^{\prime }(x^{**},y^{**})$, we substitute it into the Jacobian matrix $J^{\prime }$, where $x^{**}=\frac{C_{0}q-C_{1}q-V}{F+B(1-y)}$ and $y^{**}=\frac{S+M-F-B(1-y)}{K+M-F-B(1-y)}$; then, $J^{\prime }(E^{\prime })$ can be expressed as:

(13) $$J^{\prime }(E^{\prime })=\left[\begin{array}{l} 0\;\frac{\left(C_{0}q-C_{1}q-V)(F+B(1-y)-C_{0}q+C_{1}q+V)(K-F+M-B(1-y)+yB-B\right)}{{\left(F+B(1-y)\right)}^{2}}\\ \frac{\left(S+M-F-B(1-y))(K-S)(F+B(1-y)\right)}{{\left(K-F+M-B(1-y)\right)}^{2}}-\frac{B(S+M-F-B(1-y))(K-S)(C_{0}q-C_{1}q-V)}{{\left(K-F+M-B(1-y)\right)}^{2}(F+B(1-y))} \end{array}\right]$$

From the above expression, we can obtain Proposition 5.

**Proposition** **5.** *In scenario 1, by keeping other parameters remain unchanged, the probability of the game evolving to (1, 1) increases when the value of B (F, $C_{1}$) increases or that of S (M, $C_{0}$) decreases. In scenario 4, the probability of the private sectors choosing to provide high-quality services increases when the value of B (F) increases or the value that of S (M) decreases.*

For scenario 1, the former part of Proposition 5 is similar to Proposition 3 without the dynamic subsidy strategy. The government sectors can take some measures to make the game more likely to evolve towards the ideal result, for example, increasing the maximum subsidy amount, increasing the penalty for private sectors providing low-quality services, or improving work efficiency to reduce the supervision cost. For scenario 4, the values of DetJ at the four fixed equilibriums $A^{\prime }(0,0)$, $B^{\prime }(0,1)$, $C^{\prime }(1,0)$, and $D^{\prime }(1,1)$, are all negative. Therefore, the four equilibriums are all saddle points. Under the dynamic subsidy strategy, the game will be stable at point $E^{\prime }$. From the latter part of Proposition 5, the change of the parameters B, F, S and M will affect the coordinates of $E^{\prime }$. It helps to reduce the probability of the government sectors implementing supervision while improving the probability of the private sectors providing high-quality services when the maximum dynamic subsidy B or penalty F increases. When the cost of supervision S or the compensation for the customers M decreases, the probability of the private sectors providing high-quality services will also increases. The variation of the parameters has the same effects on the private sectors’ strategies as in scenario 1; yet, the increasing in the maximum dynamic subsidy B and penalty F help improve the probability of the private sectors providing high-quality services while reducing the probability the government sectors implementing supervision. The costs of elderly care services can also affect the probability of government sectors implementing supervision. If the constraint $F-C_{0}q+C_{1}q+V>0$ is met, the bigger the cost difference between $C_{0}$ and $C_{1}$, the higher the probability of the government sectors implementing supervision.

**Figure 5:**
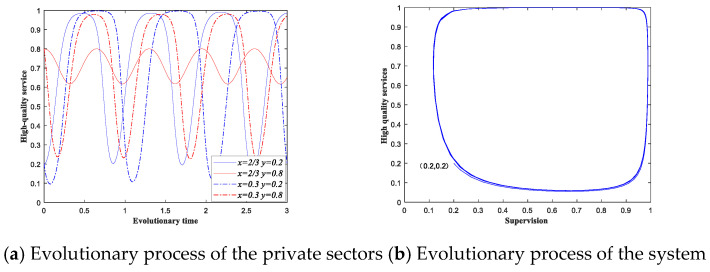
Evolutionary process of scenario 4 under static subsidy.

For a better understanding of the above conclusions, we set a numerical simulation as an example. At first, we present scenario 4 under static subsidy strategy. For scenario 4, the game cannot reach a steady state, since the four evolutionary equilibriums are all saddle points. Further, we observe the evolutionary process of scenario 4 by a numerical simulation. For example, the values of the parameters are: F=10, S=15, K=5, M=20, V=10, $C_{0}=0.3$, $C_{1}=0.2$, q=500, and θ=0.1. From these parameters, we know in scenario 4 under static subsidy strategy, the coordinates of point $\left(x^{*},y^{*}\right)$ are (2/3, 5/7). We focus on the changes of the private sectors when the probability of supervision is fixed. Figure 5a depicts the evolutionary results for the private sectors when the initial supervision percentages of the government sectors are 2/3 (solid line) and 0.3 (dotted line). The initial state of the private sectors is 0.2 and 0.8, represented by blue line and red line, respectively. When the initial state of the government sectors is fixed, the probability of the private sectors providing high-quality services fluctuates periodically over time. Figure 5b depicts the evolutionary process of the system when the initial state of the system is (0.2, 0.2). The system will not reach a stable state and the system evolution presents a periodical state.

**Figure 6:**
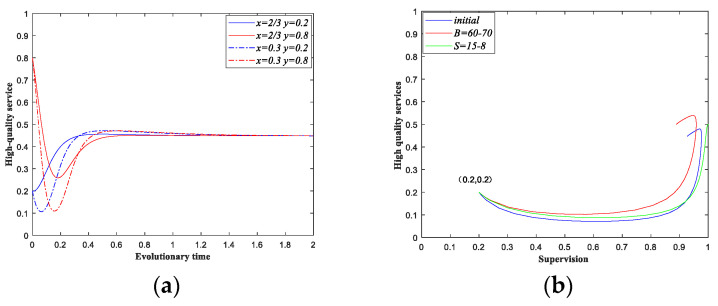
Evolutionary paths of Scenario 4 under dynamic subsidy strategy. (**a**) Evolutionary paths of the private sectors when the proportion of supervision is fixed. (**b**) Evolutionary paths of the two types of participants under the variation of parameters.

The parameter settings of scenario 4 under dynamic strategy are the same as the static subsidy strategy, moreover, we set B=60. The initial probabilities that the government sectors implement supervision are also 0.3 and 2/3. Figure 6a shows the fluctuated evolutionary path of the private sectors, and the amplitude of fluctuation decreases over time. The initial probability of the private sectors providing high-quality services has no effect on the final result and the game eventually reaches a steady state at point $E^{\prime }(x^{**},y^{**})$. The initial strategy state of the system in Figure 6b is (0.2, 0.2). Moreover, we take the changes in B and S as examples, Figure 6b shows the effects of the parameter’s variations.

From Figure 5 and Figure 6, we can see that when the government sectors implement static subsidy strategy, the system has no equilibrium between the two types of participants. When the conditions of scenario 4 under static subsidy are met, the provision process of elderly care services will show the characteristics of constant changes, and it is difficult for the system to reach a stable state. Figure 5a presents the evolutionary paths of the private sectors under different initial strategies. Compared with Figure 6a, we can see that the private sectors can reach a stable state under dynamic subsidy strategy. Figure 5b presents the evolutionary path of the system under static subsidy, while the blue line in Figure 6b show us the evolutionary path under dynamic subsidy. The system can reach a stable under dynamic subsidy strategy. Moreover, Figure 6b also presents the impacts of the parameters on the evolutionary path of the system. We can see that when the supervision cost decreases, the probability of providing high-quality services both increases. When the maximum government subsidy increases, the probability of implementing supervision decreases, and providing high-quality services both increases. Moreover, the correctness of the above analysis is verified.

## 6. Discussions

In this paper, we mainly focus on the behavioral decision-making process and results of the government sectors and private sectors in the provision process of elderly care services. Parameters like supervision cost, fine and subsidy can influence the government sectors’ supervision strategy, while the private sectors often respond to government policies, service cost and consumer demand. Moreover, each type of participants also interacts with each other in the long-term decision-making process. Therefore, this paper provides insights into how the government sectors and the private sectors make decisions under different scenarios, demand disturbance and dynamic subsidy strategy. The following (1), (2), and (3) summarize the main conclusions and answer the three questions raised in the Introduction section, respectively. Moreover, policy recommendations to promote the private sectors to provide high-quality elderly care services are also listed below.

(1) As for the government sectors, when the net revenue of the government sectors implementing supervision is positive, the government sectors choose to implement supervision strategy (the evolutionary result depends on the initial decision state of the participants in scenario 1). Otherwise, the government sectors choose to implement non-supervision strategy (scenario 2, 5, 6) or even cannot reach a stable strategy (scenario 4).

As for the private sectors, when the government sectors adopt non-supervision strategy, the private sectors have no incentive to choose to provide high quality services. When the government sectors choose to implement supervision strategy and the net revenue of providing high-quality services is positive, the private sectors will choose to provide high-quality services.

(2) The increase in subsidy for unit service and fine for providing low-quality services or the decrease in supervision cost will increase the probability the private sectors evolving towards providing high-quality services. Moreover, when the cost difference between different quality of services is less than the boundary value described in Observation 1, the increase in the cost of low-quality services or the decrease in the cost of high-quality services will promote the private sectors evolving towards providing high-quality services. (More details can be seen in the analysis of Proposition 2 and Proposition 3).

Necessary conditions exist under which the change of service demand will not affect the final decision-making of the system. When the change in service demand meets certain conditions shown in Proposition 4 (i) and (iii), the government sectors choose to implement supervision while the private sectors provide low-quality services. When the change in service demand meets the condition shown in Proposition 4 (ii), the government sectors choose to implement supervision while the private sectors provide high-quality services or the government sectors choose to implement non-supervision while the private sectors provide low-quality services, which depends on the initial decision states of the two types of participants. (Take scenario 3 as an example).

When the government sectors adopt a dynamic subsidy policy, a reasonable penalty policy for the private sectors providing low-quality services is necessary. Additionally, when certain conditions are met (see details in Observation 2), adopting the dynamic subsidy strategy is conducive to saving the government sectors’ expenditure. When the budget, penalty, and the unit cost of providing low-quality services increases or the supervision cost, the compensation to the customers and the unit cost of providing high-quality services decreases, the probability of the system evolving towards (supervision, high-quality services) increases. Moreover, the probability of the private sectors chooses to provide high-quality services increases if the penalty (the maximum government subsidy) increases or the supervision cost (compensation) decreases. In addition, the system can reach a stable equilibrium under dynamic subsidy policy, while under static subsidy policy the two types of participants are in an unstable state and cannot achieve a stable equilibrium (scenario 4).

(3) For the government sectors, if the evolutionary result is not the ideal one, the government sectors can take measures to promote the game evolving towards the ideal result. For instance, increasing penalties for low-quality services, increasing the subsidies to private service providers, reducing the supervision cost and reducing the compensation to the elder customers. These measures are all conducive to promoting the private sectors to provide high-quality elderly care services.

To promote the private sectors to provide high-quality elderly care services, we give the following policy recommendations:

Firstly, for quality assurance, the government sectors should maintain a supervision state since the private sectors may be speculative. If the government sectors implement non-supervision strategy, the private sectors will not choose to provide high-quality services because of its profit-making nature. The government sectors need to take measures to enhance the supervision efficiency; moreover, research and data collection are needed to support the formulation of related policies.

Secondly, the government sectors need to specify the minimum access standards and service standards when formulating policies related to the provision of elderly care services. Moreover, to reduce the possibility of private sectors providing low quality services, the government should increase the punishment of the private sectors for providing low-quality services. Moreover, while supervising private sectors, the government sectors can rate the private sectors and reward those with higher ratings. Integrating the prescribed service standards with the reward and punishment mechanism can ensure the implementation of the policy.

Thirdly, increasing subsidies to the private sectors promotes the provision of high-quality elderly care services. The government should increase the policy support to the private sectors. At present, construction subsidies, operating subsidies, general taxation exemptions and land allotment policies have been proposed and implemented. Policies that are more suitable for different regions and different times need to be put forward. Moreover, while strengthening supervision, the government sectors should study the behavior of private sectors. According to the actual situation, dynamic subsidy strategies can be considered when certain conditions are met, which will help the government sectors to save financial subsidy expenditures.

At last, facing the limited budget and increasing demand for elderly care services, the government sectors should establish a reasonable financial support framework for elderly care services. Moreover, the policy makers can design reasonable health promotion programs to reduce the related expenditures [11].

## 7. Conclusions

An evolutionary game model analyzing the behavioral decision of the government sectors and the private sectors in the provision process of elderly care services is established. Firstly, eight possible evolution scenarios and five possible evolutionary results are given. Then, the necessary conditions to obtain different evolutionary results are summarized and the influence of different factors on the system are analyzed. Secondly, the impact of demand disturbance on the system are analyzed. At last, for a more comprehensive and systematic analysis of the provision process of elderly care service system, we also explore the interaction mechanism between the government sectors and the private sectors under dynamic subsidy policy. Numerical simulations are given to verify our conclusions and make our model analysis easier to understand. The evolutionary model is beneficial to identify the decision-making behaviors of the government sectors and the private sectors. Moreover, the model can not only help the government sectors to formulate relevant policies, but also lead the private sectors in strategy choices under different scenarios and dynamic situation. It can also help the government sectors to establish a feasible long-term care system to meet the growing demand of elderly care services in many countries.

There are still some improvements to be made of this paper. Firstly, only two types of participants are considered in the provision process of elderly care services. However, the service receiver (the elderly) can also be considered which will make the model analysis deeper. Moreover, most theoretical articles in the field of operation management contain strong hypotheses, the similar problem in our paper is not inevitable. Further, part of the numerical experimental data derived from empirical estimate. These issues are to be addressed in future research.

## Figures and Tables

**Table 1:** Variables and definitions.

Variables	Meaning of Variables
R	Earnings of the government sectors when providing high-quality services, R>0
S	Supervision cost of the government sectors when implementing supervision, S>0
θ	Unit subsidy for providing elderly care services, θ>0
K	Additional earnings when the government sectors implement supervision and the private sectors provide high-quality services, K>0
F	Fine imposed on the private sectors for low-quality services when the government sectors implement supervision, F>0
M	Compensation paid to customers when the government sectors implement supervision but the private sectors provide low-quality services
x,1−x	Probability that the government sectors implement supervision/non-supervision, 0<x<1
y,1−y	Probability that the private sectors provide high/low quality services, 0<y<1
$E_{g1},E_{g2},\bar{E_{g}}$	Supervision/non-supervision/group average returns of the government sectors
$E_{s1},E_{s2},\bar{E_{s}}$	High-quality services/low-quality services/group average returns of the private sectors
P	Unit price of elderly care services, P>0
q	Demand for elderly care services, q>0
Δq	Change in demand for elderly care services
$C_{0}$, $C_{1}$	Unit cost of high-quality/low-quality elderly care services, $C_{0}>C_{1}>0$
V	Risk of supplying low-quality services, V>0

**Table 2:** Payoff matrix of government sectors and private sectors.

		Private Sectors	
		Hs (y)	Ls (1−y)
**Government sectors**	Is (x)	Is: *R* − *S* − *θq* + *K*	Is: −*S* + *F* − *M*
		Hs: *P_q_* − *C*_0_*q* + *θq*	Ls: *P_q_* − *C*_1_*q* − *F* − *V*
	Ns (1−x)	Ns: *R* − *θq*	Ns: −*θq*
		Hs: *P_q_* − *C*_0_*q* + *θq*	Ls: *P_q_* − *C*_1_*q* + *θq* − *V*

**Table 3:** Expressions of DetJ and TrJ at the evolutionary equilibriums.

EE	DetJ	TrJ
*A*(0,0)	*π*_2_·*π*_4_	*π*_2_+*π*_4_
*B*(0,1)	−*π*_2_·*π*_3_	−*π*_2_+*π*_3_
*C*(1,0)	−*π*_4_·*π*_1_	−*π*_4_+*π*_1_
*D*(1,1)	−*π*_1_·(−*π*_3_)	−*π*_1_−*π*_3_

Note: EE: evolutionary equilibrium.

**Table 4:** Local stability of evolutionary equilibriums.

**EE**	**Scenario 1**			**Scenario 2**			**Scenario 3**			**Scenario 4**		
	**D**	**T**	**S**	**D**	**T**	**S**	**D**	**T**	**S**	**D**	**T**	**S**
A (0, 0)	+	-	ESS	+	-	ESS	-	Uc	Sa	-	Uc	Sa
B (0, 1)	+	+	Us	-	Uc	Sa	+	+	Us	-	Uc	Sa
C (1, 0)	+	+	Us	+	+	Us	-	Uc	Sa	-	Uc	Sa
D (1, 1)	+	-	ESS	-	Uc	Sa	+	-	ESS	-	Uc	Sa
E (x*, y*)	-	0	Sa	/	/	/	/	/	/	+	0	Sa
**EE**	**Scenario 5**			**Scenario 6**			**Scenario 7**			**Scenario 8**		
	**D**	**T**	**S**	**D**	**T**	**S**	**D**	**T**	**S**	**D**	**T**	**S**
A (0, 0)	+	-	ESS	+	-	ESS	-	Uc	Sa	-	Uc	Sa
B (0, 1)	+	+	Us	-	Uc	Sa	+	+	Us	-	Uc	Sa
C (1, 0)	-	Uc	Sa	-	Uc	Sa	+	-	ESS	+	-	ESS
D (1, 1)	-	Uc	Sa	+	+	Us	-	Uc	Sa	+	+	Us

Note: “/”: inexistence; D: *DetJ*; T: *TrJ*; S: Stability; Us: Unstable point; Uc: uncertain; Sa: Saddle point.

**Table 5:** Parameter settings for two initial situations.

	F	$C_{0}$	$C_{1}$	q	S	M	V	K	θ
(1)	3	0.3	0.2	300	8	30	10	20	0.1
(2)	10	0.3	0.2	500	10	20	10	5	0.1

**Table 6:** Expressions of DetJ and TrJ at the evolutionary equilibriums under dynamic subsidy.

EE	DetJ	TrJ
$A^{\prime }(0,0)$	$\left(F-S-M+B(1-y))(-C_{0}q+C_{1}q+V\right)$	$\left(F-S-M+B(1-y))+(-C_{0}q+C_{1}q+V\right)$
$B^{\prime }(0,1)$	$\left(K-S)(C_{0}q-C_{1}q-V\right)$	$K-S+C_{0}q-C_{1}q-V$
$C^{\prime }(1,0)$	$-(F-S-M+B(1-y))(F+B(1-y)-C_{0}q+C_{1}q+V)$	$-(F-S-M+B(1-y))+(F+B(1-y)-C_{0}q+C_{1}q+V)$
$D^{\prime }(1,1)$	$\left(K-S)(F-C_{0}q+C_{1}q+V\right)$	$-(K-S)-(F-C_{0}q+C_{1}q+V)$

**Figure A1:**
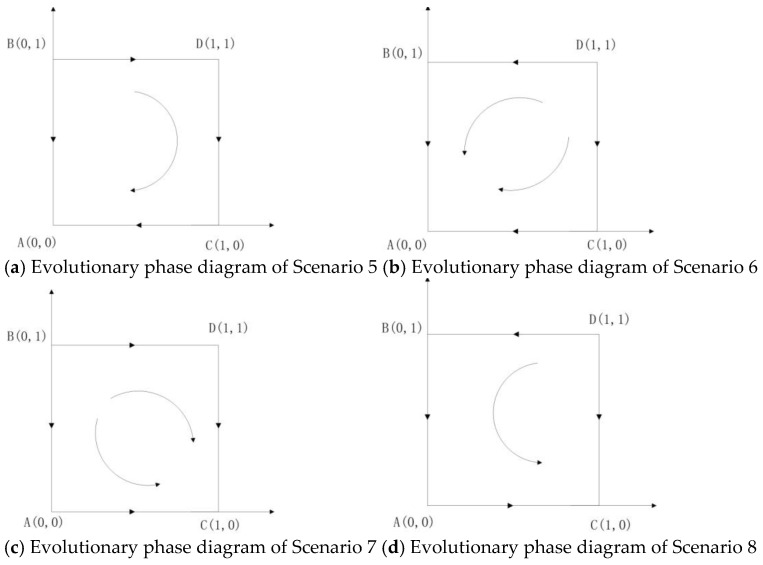
Evolutionary phase diagrams of Scenario 5 to 8.

**Figure A2:**
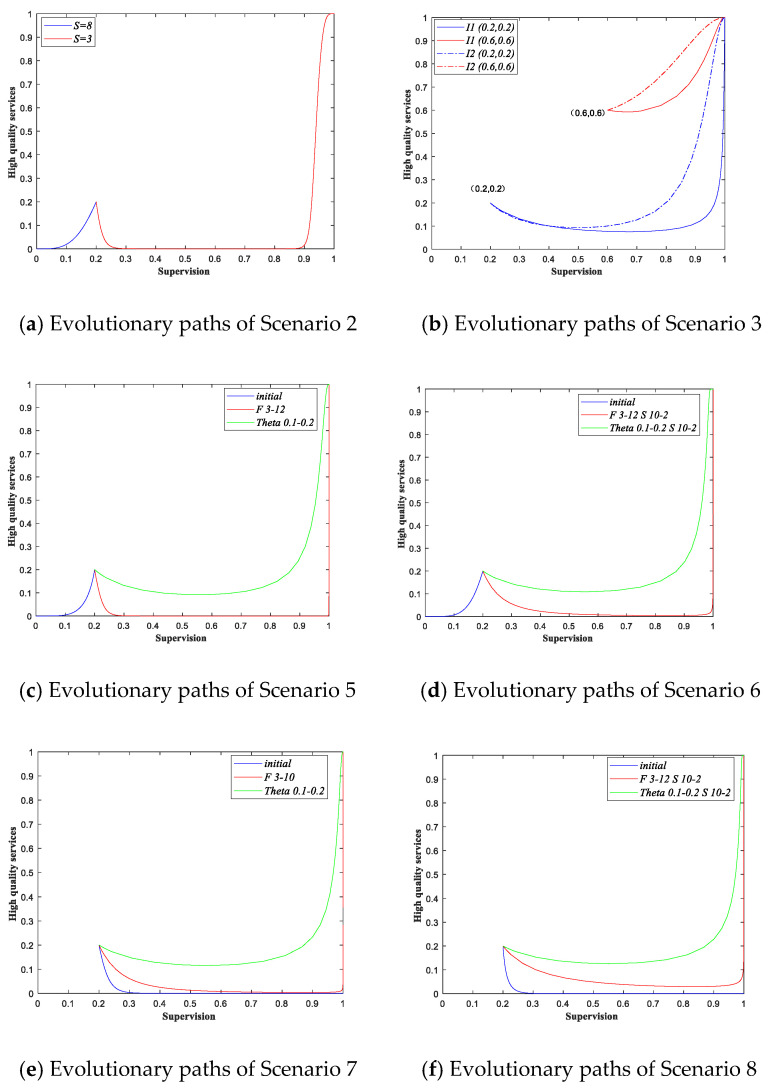
The evolutionary paths of the system in different scenarios.

## Appendix A

**Scenario** **5.**
*When $\pi_{1}<0$, $\pi_{2}<0$, $\pi_{3}>0$, $\pi_{4}<0$ are satisfied, the evolutionary stable strategy of the game is (0, 0). In this case, regardless of the government strategy, the net benefit of providing high-quality services is negative. Hence, the private sectors will choose to provide low-quality services. Though the net benefits of supervision are positive when the private sectors choose to provide high-quality services, the strategy of the government sectors evolve towards non-supervision since the private sectors is bound to provide low-quality services. The government sectors will implement non-supervision and the private sectors will provide low-quality services eventually.*

**Scenario** **6.**
*When $\pi_{1}<0$, $\pi_{2}<0$, $\pi_{3}<0$, $\pi_{4}<0$ are satisfied, the evolutionary stable strategy of the game is (0, 0). The net benefits of implementing supervision are negative regardless the private sectors’ strategy. Meanwhile, for the private sectors, the net benefits of providing high-quality services are negative regardless the government’s strategy. The system will evolve towards non-supervision, low quality services.*

**Scenario** **7.**
*When $\pi_{1}<0$, $\pi_{2}<0$, $\pi_{3}>0$, $\pi_{4}>0$ are satisfied, the evolutionary stable strategy of the game is (1, 0). In this case, regardless of the government strategy, the net benefit of providing high-quality services is negative. For the government sectors, the net benefits of implementing supervision are always positive. The government sectors will choose to implement supervision while the private sectors will choose to provide low-quality services eventually.*

**Scenario** **8.**
*When $\pi_{1}<0$, $\pi_{2}<0$, $\pi_{3}<0$, $\pi_{4}>0$ are satisfied, the evolutionary stable strategy of the game is (1, 0). In this case, the net benefit of providing high-quality services is negative, regardless of the government strategy. For the private sectors, it will evolve towards providing low-quality services. For the government sectors, the net benefit of implementing supervision is positive when the private sectors provide low-quality services. Therefore, the evolutionary result of the system is supervision-low quality services.*

The phase diagrams of the four scenarios are shown in Figure A1 below.

## Appendix B

**1.** **Proof** **of** **Proposition** **1.** The equilibrium points of the replicator dynamic system can be obtained from: F(x)=0, F(y)=0, where (x,y)∈[0,1]×[0,1]. Therefore, part (i) is clear in that the four fixed points (0, 0), (1, 0), (0, 1), (1, 1) are equilibriums that can satisfy equations F(x)=0, F(y)=0. For part (ii), point $\left(x^{*},y^{*}\right)$ is an equilibrium point only if equations y(K−F+M−θq)+F−S−M+θq=0 and $x(F+\theta q)-C_{0}q+C_{1}q+V=0$ hold, where $\left(x^{*},y^{*})\in [0,1]\times [0,1\right]$. For $\left(x^{*},y^{*})\in [0,1]\times [0,1\right]$, we get that $0\leq C_{0}q-C_{1}q-V\leq F+\theta q$ and when S≤K then S+M−F−θq≥0 or when K≤S then S+M−F−θq≤0. Part (ii) is proven. □

**2.** **Proof** **of** **Proposition** **2.** We can obtain the ESS of each scenario easily from Table 4 according to Friedman (1991). Taking (i) as an example, when conditions $\pi_{1}>0$ and $\pi_{3}>0$ are met, we have $F+\theta q-C_{0}q+C_{1}q+V>0$ and K>S. According to the expressions of DetJ and TrJ at equilibrium (1, 1) in Table 3, we can obtain DetJ>0 and TrJ<0 at point (1, 1). Similarly, when $\pi_{4}<0$, we have F−S−M+θq<0. According to the expressions of DetJ and TrJ at equilibrium point (0, 0), we can get DetJ>0 and TrJ<0 at point (0, 0). Part (iii) can be proven in the same way. □

**3.** **Proof** **of** **Observation** **1.** From Table 4, in scenarios 1 and 3, the ESS may be (1, 1). That is, when the conditions $\pi_{1}>0$ and $\pi_{3}>0$ are met, the game will eventually evolve to (1, 1) (in scenario 1, the eventual evolutionary results are also related to the initial state and parameter values). Therefore, when $F+\theta q-C_{0}q+C_{1}q+V>0$ is met, the cost difference satisfies $C_{0}-C_{1}<\frac{F+\theta q+V}{q}$. □

**4.** **Proof** **of** **Proposition** **3.** Taking the partial derivation of $S_{BECD}$ with respect to parameters and according to the constraints satisfied by scenario 3, we can get $\frac{\partial S_{BECD}}{\partial C_{1}}=\frac{q}{2(F+\theta q)}>0$, $\frac{\partial S_{BECD}}{\partial C_{0}}=-\frac{q}{2(F+\theta q)}<0$. Similarly, we can get $\frac{\partial S_{BECD}}{\partial F}=\frac{C_{0}q-C_{1}q-V}{2{\left(F+\theta q\right)}^{2}}+\frac{K-S}{2(K-F+M-\theta q)}>0$, $\frac{\partial S_{BECD}}{\partial \theta }=\frac{q\cdot (K-S)}{2{\left(K-F+M-\theta q\right)}^{2}}>0$ and $\frac{\partial S_{BECD}}{\partial q}=\frac{F\cdot (-C_{0}+C_{1})-2\theta q}{2{\left(F+\theta q\right)}^{2}}<0$, $\frac{\partial S_{BECD}}{\partial S}=-\frac{1}{2(K-F+M-\theta q)}<0$, and $\frac{\partial S_{BECD}}{\partial M}=\frac{S-K}{2{\left(K-F+M-\theta q\right)}^{2}}<0$. We have already known that the area of $S_{BECD}$ represents the probability that the evolutionary result is (1, 1). Proposition 3 is proved. □

**5.** **Proof** **of** **Proposition** **4.** We know that, when the conditions DetJ>0 and TrJ<0 are met, the equilibrium point is the ESS of the game. Therefore, when the conditions in part (i) are met, we have $F+\theta (q+\Delta q)-C_{0}(q+\Delta q)+C_{1}(q+\Delta q)+V<0$ and F+θ(q+Δq)−S−M>0. Conditions DetJ>0 and TrJ<0 are met at equilibrium point (1, 0). As such, part (i) is proven. □

Similarly, when the conditions in part (ii) are met, we have F+θ(q+Δq)−S−M<0 and $F+\theta (q+\Delta q)-C_{0}(q+\Delta q)+C_{1}(q+\Delta q)+V>0$. Conditions DetJ>0 and TrJ<0 are met at equilibriums (0, 0) and (1, 1). For (iii), we have $F+\theta (q+\Delta q)-C_{0}(q+\Delta q)+C_{1}(q+\Delta q)+V<0$, and equilibrium point (1, 0) is the ESS.

**6.** **Proof** **of** **Proposition** **5.** The results of the dynamic subsidy policy in scenario 1 are similar to Proposition 3. Taking the partial derivation of $S_{BECD}$ with respect to these related parameters, we can get $\frac{\partial S_{BECD}}{\partial C_{1}}=\frac{q}{2\left[F+B(1-y)\right]}>0$, $\frac{\partial S_{BECD}}{\partial C_{0}}=-\frac{q}{2\left[F+B(1-y)\right]}<0$, $\frac{\partial S_{BECD}}{\partial F}=\frac{C_{0}q-C_{1}q-V}{2{\left[F+B(1-y)\right]}^{2}}+\frac{K-S}{2{\left[K-F+M-B(1-y)\right]}^{2}}>0$, $\frac{\partial S_{BECD}}{\partial S}=-\frac{1}{2[K-F+M-B(1-y)]}<0$, $\frac{\partial S_{BECD}}{\partial B}=\frac{\left(1-y)(C_{0}q-C_{1}q-V\right)}{2{\left[F+B(1-y)\right]}^{2}}+\frac{\left(1-y)\cdot (K-S\right)}{2{\left[K-F+M-B(1-y)\right]}^{2}}>0$, $\frac{\partial S_{BECD}}{\partial M}=\frac{S-K}{2{\left[K-F+M-B(1-y)\right]}^{2}}<0$. Therefore, the first part of proposition 5 are proved. For scenario 4, under dynamic subsidy policy, the system is asymptotically stable at point $\left(x^{**},y^{**}\right)$. When the value of $y^{**}$ increases the probability of the private sectors choosing to provide high-quality services increases. Then, we take the partial derivation of $x^{**}$ and $y^{**}$ with respect to these related parameters. We can thus get $\frac{\partial x^{**}}{\partial C_{0}}=\frac{q}{F+B(1-y)}>0$, $\frac{\partial x^{**}}{\partial C_{1}}=-\frac{q}{F+B(1-y)}<0$, $\frac{\partial x^{**}}{\partial F}=\frac{-C_{0}q+C_{1}q+V}{{\left[F+B(1-y)\right]}^{2}}<0$, $\frac{\partial x^{**}}{\partial B}=\frac{\left(1-y)\cdot (-C_{0}q+C_{1}q+V\right)}{{\left[F+B(1-y)\right]}^{2}}<0$, $\frac{\partial y^{**}}{\partial F}=\frac{-K+S}{{\left[K+M-F-B(1-y)\right]}^{2}}>0$, $\frac{\partial y^{**}}{\partial B}=\frac{\left(-K+S)(1-y\right)}{\left[K+M-F-B(1-y)\right]}>0$, $\frac{\partial y^{**}}{\partial S}=\frac{1}{K+M-F-B(1-y)}<0$ and $\frac{\partial y^{**}}{\partial M}=\frac{K-S}{{\left[K+M-F-B(1-y)\right]}^{2}}<0$. Then, the second part of Proposition 5 is proved. □

## Appendix C

Parameters are derived from local policies and regulations in China in this paper, as well as practical cases of private elderly care institutions. Specifically, for instance, ‘Regulations of Zhejiang Province on the Promotion of social elderly care services’ impose a fine of CNY 30,000 to 100,000 on institutions that do not meet the requirements. Without loss of generality, we define the value range of the fine as CNY 10,000 to 150,000. Generally speaking, number of people served by a single private elderly care service institution range from 100 to 1500 (medium-sized institutions are more common). The unit price of private elderly care services ranges from CNY 1000 to 9000 per month. We assume that the average price is CNY 5000. Thus, we assign the cost of high-quality elderly care services as CNY 3000 to 3500, and the cost of low-quality elderly care services as CNY 1000 to 2500. The unit subsidy price varies with the level of development of the region. In most regions, the unit subsidy is generally CNY 1000 to 4000. In addition, we estimate the value range of the following parameters based on relevant cases, references and experience. For *S*, ranging from CNY 10,000 to 150,000. For *V*, ranging from CNY 100,000 to 300,000. For *K*, ranging from CNY 50,000 to 300,000. For *M*, ranging from CNY 100,000 to 300,000. We define a unit as CNY ten-thousand and the unit of *q* is the number of people.

## Appendix D

In Section 3.3, we take scenario 1 and scenario 4 as examples to analyze the evolutionary path of the system and the influence of parameter changes on the evolutionary results of the system. To save the body context space, numerical experiments of the remaining scenarios are listed in Table A1 and Figure A2 below.

**Table A1:** Parameter settings for six initial situations.

	F	$C_{0}$	$C_{1}$	q	S	M	V	K	θ
Scenario 2	3	0.3	0.2	300	8	30	10	5	0.1
Scenario 3 (I1)	10	0.3	0.2	500	10	20	10	20	0.1
Scenario 3 (I2)	10	0.3	0.2	300	10	20	10	20	0.1
Scenario 5	3	0.3	0.1	300	10	30	25	20	0.1
Scenario 6	3	0.3	0.1	300	10	30	25	5	0.1
Scenario 7	3	0.3	0.15	300	10	20	10	20	0.1
Scenario 8	3	0.3	0.15	300	10	20	10	5	0.1

From the numerical experiments above, the conclusions in this paper are verified. For example, in Figure A2a, in the initial state, the system reaches a stable state at (0, 0). Since the scenario 2 met the conditions $\pi_{1}>0$ and $\pi_{3}<0$, when the supervision cost decreases until the conditions $\pi_{3}>0$ is met, then the system evolves towards the ideal result (1, 1). In Figure A2b, we show two initial strategy state (0.2, 0.2) (0.6, 0.6) and two sets of parameters. We can see that higher proportion of initial strategy states makes the system evolve to the ideal result faster. In Figure A2c–f, we can see that by adjusting the related parameters, the system can meet the conditions to obtain the ideal equilibrium result (1, 1).
